# Supplementary material for: Epigenetic rewriting at centromeric DNA repeats leads to increased chromatin accessibility and chromosomal instability
Source: Epigenetics Chromatin. 2021 Jul 28;14:35. doi: 10.1186/s13072-021-00410-x (PMC8317386; doi:10.1186/s13072-021-00410-x)
Supplement: Supplementary file 1 — Additional file 1: Figure S1. Visualization of the TALE fusions proteins and CENP-A. U2OS cells expressing either the TALE-demethylase (top), its point mutant (middle) or the TALE-GFP (bottom). TALE proteins are visualized using an anti-HA antibody (shown in green), CENP-A is revealed with an anti-CENP-A antibody (shown in red) and DNA was stained using Hoechst. Maximum projections are shown for each cell. Scale bar, 10 μm. Figure S2. Characterization of the overlap of TALE-fusion proteins with CENP-A and D7Z1. Percentage of overlap between TALE and CENP-A foci (left, n=277 foci) and TALE and D7Z1 foci (right, n=316 foci). The value presented is the volume of the TALE covered by the other structure (in %). Figure S3. Chromosome instability in TALE-demethylase cell line. (A) The volumes of nuclei are represented according to the number of chromosomes 11 (top two panels, green) or 7 (bottom two panels, orange) in the inducible cell line expressing the TALE-KMD4B, with or without doxycycline treatment. The muted area around the solid line represents the standard error while the black dots are the individual data points (nuclei volume) for each number of chromosomes. (B) The number of chromosomes 7 and 11 are determined in a thousand nuclei of the TALE-KDM4B cell line, after 48 h of growth with or without doxycycline treatment. The histogram represents the number of nuclei counted according to the number of chromosomes 7 (green) or 11 (orange) in each nucleus. Figure S4. Effects of TALE-demethylase expression on CENP-A loading. (A) Boxplots showing the signal intensity (left panel) and the volume (right panel) of CENP-A foci associated with the TALE-demethylase foci (blue) (n=186 foci) or with the TALE-KDM4B-H188A foci (orange) (n=82). (B) Boxplot showing the percentage of overlap of TALE-demethylase foci by CENP-A foci (blue) (n=186 foci) and the overlap of TALE-KDM4B-H188A foci by CENP-A foci (orange) (n=82). The value presented is the percentage of the volume of the [file 13072_2021_410_MOESM1_ESM.pdf]

# Supplemental information

Decombe *et al.*

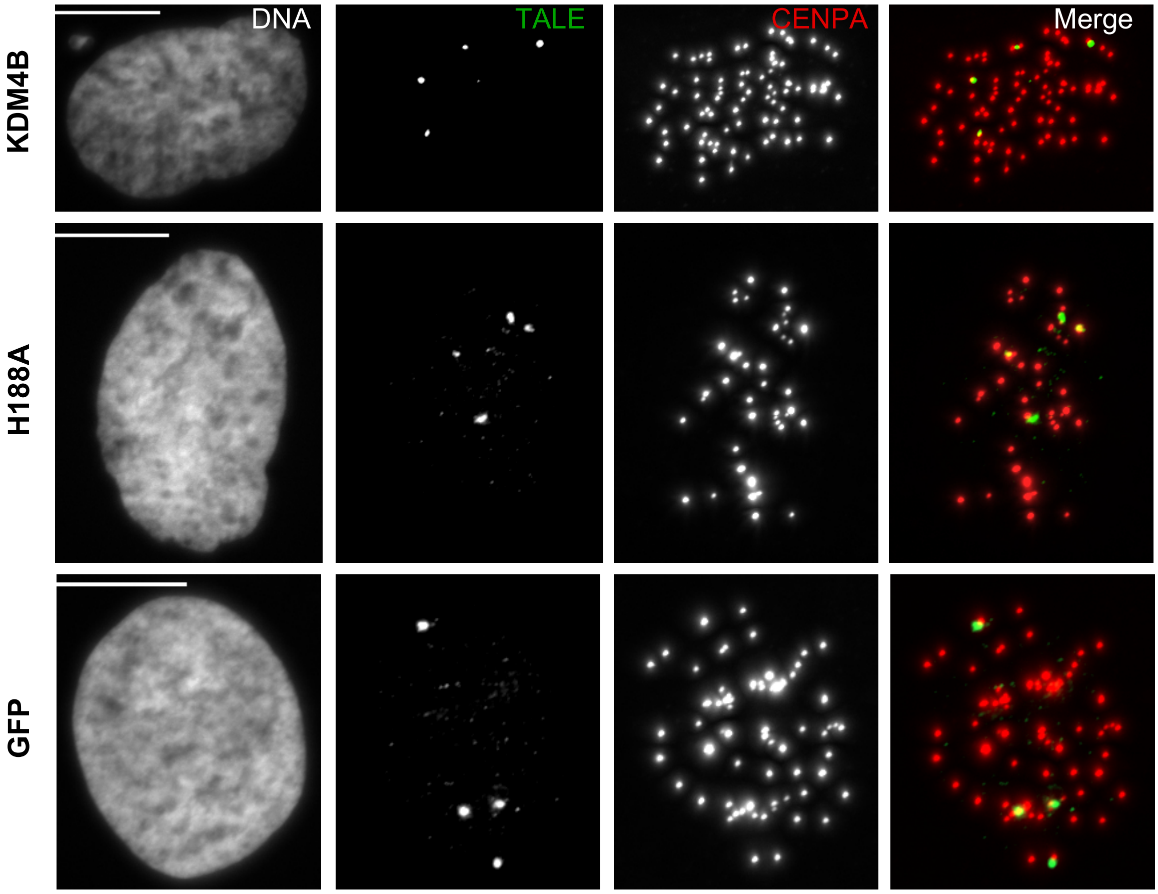


**Supplemental Figure 1: Visualization of the TALE fusions proteins and CENP-A**

U2OS cells expressing either the TALE-demethylase (top), its point mutant (middle) or the TALE-GFP (bottom). TALE proteins are visualized using an anti-HA antibody (shown in green), CENP-A is revealed with an anti-CENP-A antibody (shown in red) and DNA was stained using Hoechst. Maximum projections are shown for each cell. Scale bar, 10 µm.

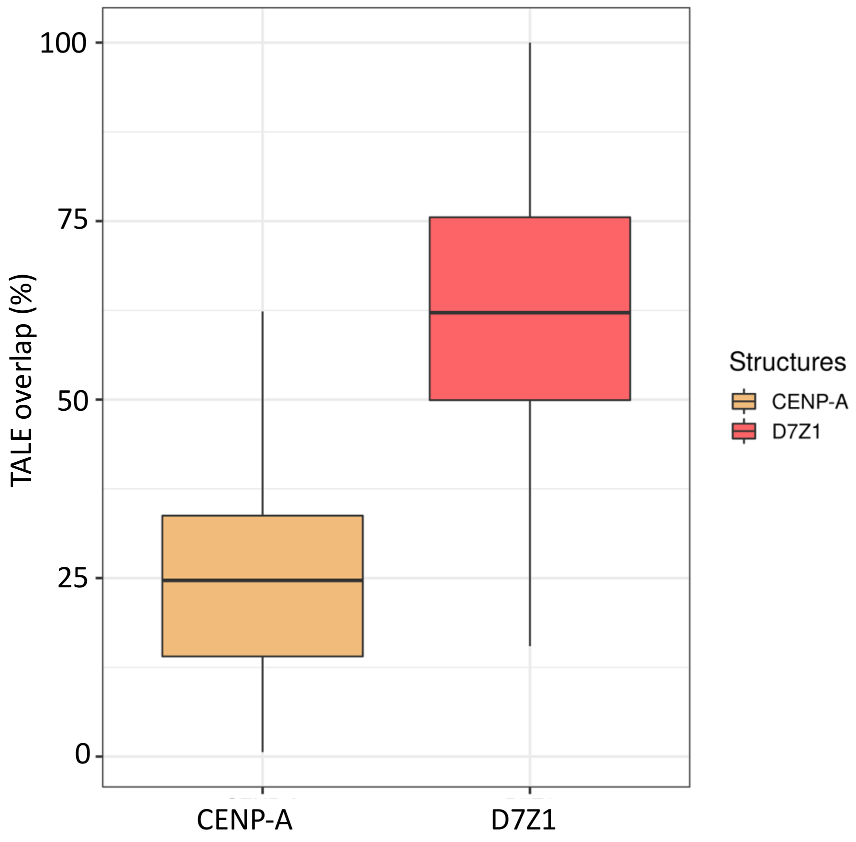


**Supplemental Figure 2: Characterization of the overlap of TALE fusion proteins with CENP-A and D7Z1**

Percentage of overlap between TALE and CENP-A foci (left, n=277 foci) and TALE and D7Z1 foci (right, n=316 foci). The value presented is the volume of the TALE covered by the other structure (in %).

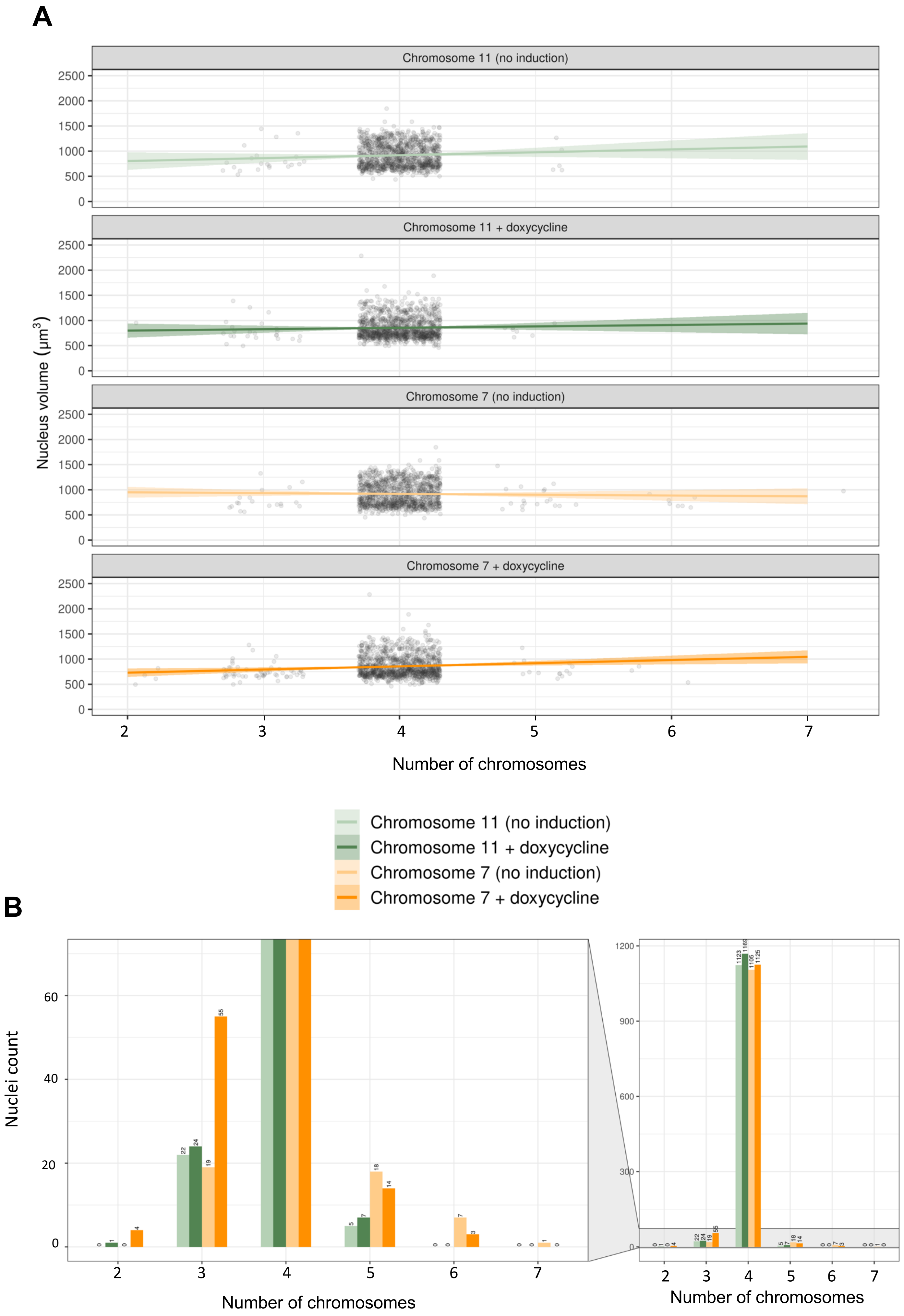


**Supplemental Figure 3**

**Supplemental Figure 3: Chromosome instability in TALE-demethylase cell line**

(**A**) The volumes of nuclei are represented according to the number of chromosomes 11 (top two panels, green) or 7 (bottom two panels, orange) in the inducible cell line expressing the TALE-KMD4B, with or without doxycycline treatment. The muted area around the solid line represents the standard error while the black dots are the individual data points (nuclei volume) for each number of chromosomes. (**B**) The number of chromosomes 7 and 11 are determined in a thousand nuclei of the TALE-KDM4B cell line, after 48h of growth with or without doxycycline treatment. The histogram represents the number of nuclei counted according to the number of chromosomes 7 (green) or 11 (orange) in each nucleus.

A

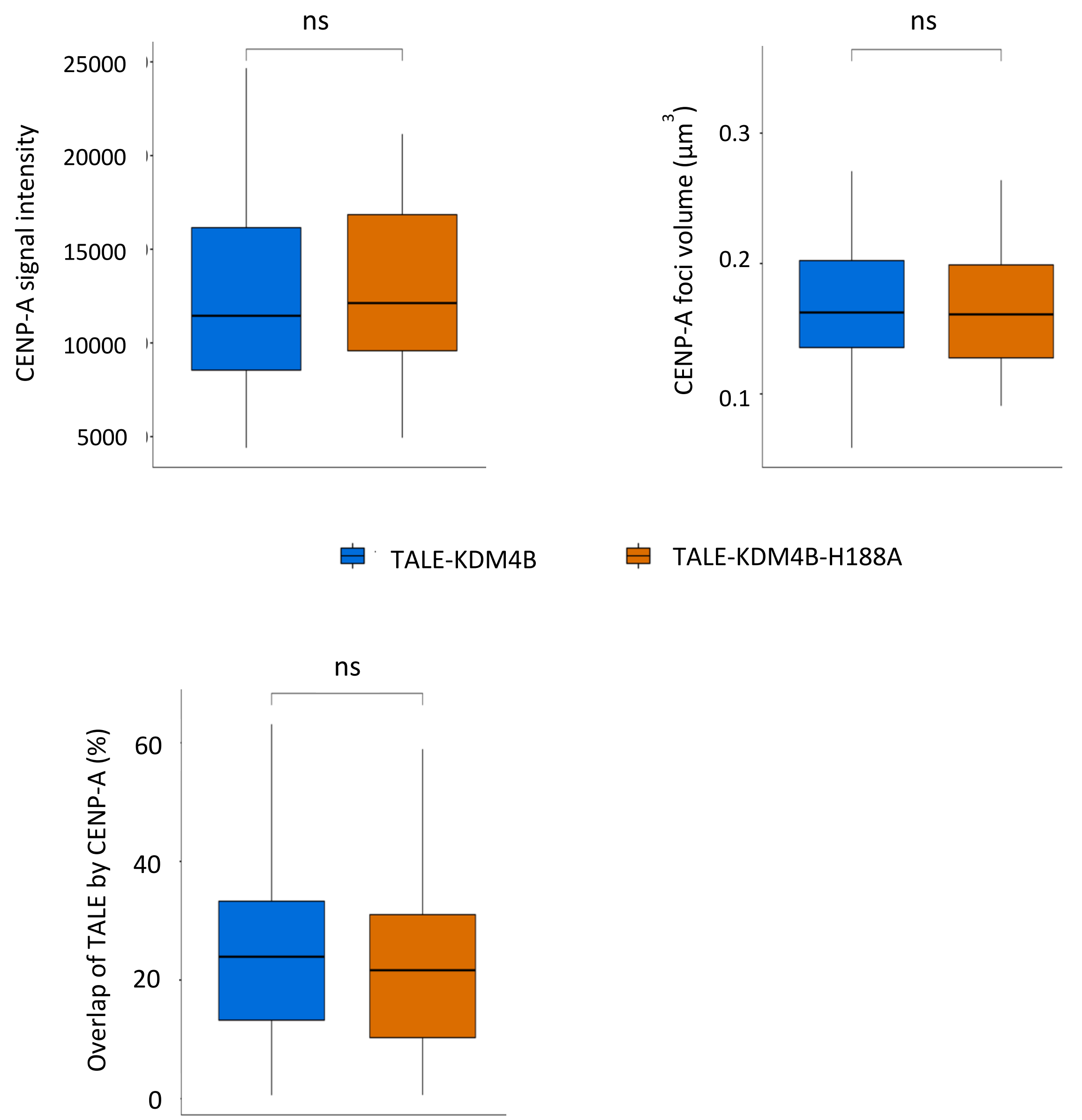


**Supplemental Figure 4: Effects of TALE-demethylase expression on CENP-A loading**

(A) Boxplots showing the signal intensity (left panel) and the volume (right panel) of CENP-A foci associated with the TALE-demethylase foci (blue) (n=186 foci) or with the TALE-KDM4B-H188A foci (orange) (n=82). (**B**) Boxplot showing the percentage of overlap of TALE-demethylase foci by CENP-A foci (blue) (n=186 foci) and the overlap of TALE-KDM4B-H188A foci by CENP-A foci (orange) (n=82). The value presented is the percentage of the volume of the TALE covered by CENP-A.

| | *TALE-KDM4B* | | | | *TALE-GFP* | | | |
|---|---|---|---|---|---|---|---|---|
| | *Chr. 7* | | *Chr. 11* | | *Chr. 7* | | *Chr. 11* | |
| *Doxyxycline* | + | - | + | - | + | - | + | - |
| *Nuclei with 4 chr.* | 1125 *(93.7%)* | 1105 *(96.1%)* | 1169 *(97.3%)* | 1123 *(97.7%)* | 959 *(98.2%)* | 1069 *(98.3%)* | 954 *(97.6%)* | 1068 *(98.2%)* |
| *Nuclei with +/- 4 chr.* | 76 *(6.3%)* | 45 *(3.9%)* | 32 *(2.7%)* | 27 *(2.3%)* | 18 *(1.8%)* | 19 *(1.7%)* | 23 *(2.4%)* | 20 *(1.8%)* |
| *Total* | 1201 | 1150 | 1201 | 1150 | 977 | 1088 | 977 | 1088 |

**Additional Table 1: Chromosome instability upon removal of chromosome 7 pericentromeric H3K9me3.** Number of nuclei containing either 4 chromosomes 7 and 11 or a different number. Nuclei containing more or less than 4 chromosomes are considered products of aberrant mitosis and proof of genomic instability. Cells grown with doxycycline for 48h (+) are expressing either the TALE-demethylase or the TALE-GFP (control), while cells grown without (-) do not express any construct.

| **Name** | **Promoter** | **Insert** | **Size** (bp) |
|---|---|---|---|
| | | **Transient transfections** | |
| pJL170 | CMV | FLAG-TALE 394 | 5973 |
| pJL179 | CMV | HA-TALE 394 + GFP | 6183 |
| pJL193 | CMV | HA-TALE 394 + KDM4B | 8763 |
| pJL195 | CMV | HA-TALE394 + KDM4B-H188A | 8763 |
| pJL203 | CMV | HA-TALE 394 + KDM4B + Dendra2 | 9501 |
| pJL206 | CMV | HA-TALE394 + KDM4B-H188A + Dendra2 | 9501 |
| | | **Stable cell lines** | |
| pJL204 | pTRE3G | HA-TALE 394 + KDM4B | 8560 |
| pJL227 | pTRE3G | HA-TALE 394 + GFP | 5999 |

**Additional Table 2: Plasmids used in this study**
